# Treatment of Failed Proximal Femoral Nail Anti-rotation Asia (PFNA2) in a Severely Osteoporotic Patient With a Revision Stem

**DOI:** 10.7759/cureus.55152

**Published:** 2024-02-28

**Authors:** Amit Kale, Rahul Salunkhe, Faiz Rahman Pervez, Ishan Shevate, Pankaj Sharma

**Affiliations:** 1 Department of Orthopaedics, Dr. D. Y. Patil Medical College, Hospital & Research Centre, Pune, IND

**Keywords:** internal fixation, bipolar hemiarthroplasty, proximal femoral nail antirotation, osteoporosis, intertrochanteric fracture

## Abstract

An intertrochanteric fracture is a prevalent and perilous kind of fracture that often affects older persons. A customized implant, proximal femoral nail anti-rotation Asia (PFNA2) is being used expressly in unstable intertrochanteric fractures in people with osteoporosis. In this case report, we examined a female osteoporosis patient, age 74, who underwent a failed PFNA2 procedure. Subsequently, the patient had bipolar hemiarthroplasty as a treatment. To prevent mechanical failure, it is crucial to strive for a high level of reduction quality and precise alignment of the central blade throughout hip X-ray procedures. Improved surgical proficiency and skill are crucial for managing patients with severe osteoporosis and prolonged weight-bearing requirements, hence reducing the occurrence of postoperative problems. Depending on the cause of the failure and the individual circumstances of the patient when internal fixation fails, it is recommended to either replace the joint with a prosthetic or reapply fixation. These interventions may facilitate the production of beneficial healing outcomes.

## Introduction

Intertrochanteric (IT) fractures in the elderly pose a frequent and significant task for orthopedic surgeons. There is a higher susceptibility to osteoporosis and resulting hip fractures in the Indian population [1]. In one study done in India, 50% of women and 36% of men above the age of 50 had low bone mass [2]; hip fractures are frequent among this age group, with IT fractures accounting for 50% of hip fractures in older individuals, more than half of which are defined as unstable [3].

A plethora of comparative studies in the literature have evaluated the efficiency of extramedullary vs. intramedullary implants in IT fracture fixation. Intramedullary fixation devices and sliding compression hip screws with a side-plate assembly, such as the dynamic hip screw (DHS), are the two main types of internal fixation devices that are typically used for IT fractures. Stable IT fractures are commonly treated with the compression hip screw [4]. However, this screw type has a higher likelihood of cut-out failure (6%-19%) in instances involving unstable IT fractures (AO types 31A2 and 31A3) [5,6].

Introduced in 2003, proximal femoral nail anti-rotation (PFNA), a variant of the proximal femoral nail (PFN), is characterized by the integration of a spiral blade. Several published studies have demonstrated the clinical advantages of the PFNA compared to the DHS in stabilizing IT fractures [7]. Recently, several orthopedic surgeons have begun advocating the use of the newer Proximal Femoral Nail Anti-rotation Asia (PFNA2) implant over the DHS to treat stable IT fractures in older patients [8]. Despite the potential benefits of the PFN for treating unstable fractures in the elderly, problems such as screw cut-out and fracture fixation failure are common in this age group because of osteoporosis. Mechanical stability is also reduced because of impaired bone quality [9].

The superiority and therapeutic benefits of these more modern implants in stable IT fractures have not been proven owing to a lack of appropriate comparative trials that exclusively involve stable fractures. Substantial issues were observed when the PFNA was employed in Asian patients [10]. Consequently, the AO/Association of the Study of Internal Fixation (ASIF) designed a new type of nail called the PFNA2, which is specifically suited for the form of the femur in Asian people [11]. Bipolar hemiarthroplasty results in earlier mobilization as compared to PFN.

## Case presentation

**Figure 1:**
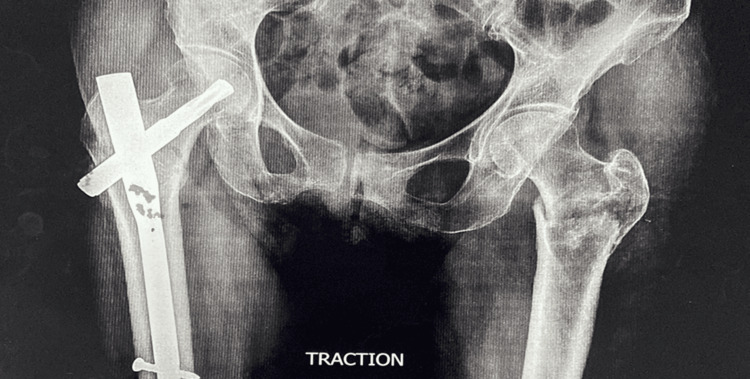
Plain radiograph of the pelvis with hips showing an operated case of a left intertrochanteric fracture with in situ PFNA2 and right-sided intertrochanteric fracture (Boyd and Griffin classification type 1) PFNA2: proximal femoral nail anti-rotation Asia

A 74-year-old female patient presented to our emergency room with discomfort in her left hip and difficulty walking after a reported slip and fall at home. A standard X-ray of the pelvis, with both hips in an anterior-posterior perspective, showed a fracture in the left IT region of the femur (Figure 1).

The patient previously had a right-sided IT fracture, which was treated with PFNA2 two months prior. The patient currently does not have any new symptoms or complaints. The patient was released with instructions to avoid putting any weight on the affected area and did not have any notable problems after the surgery.

All routine blood investigations were within normal limits. A computed tomography (CT) scan of the patient’s pelvis, abdomen, and hips was performed, which showed a fracture in the right IT region. The patient’s bone mineral density (BMD) score was 0.630 g/cm^2^, indicating severe osteoporosis according to World Health Organization categorization.

**Figure 2:**
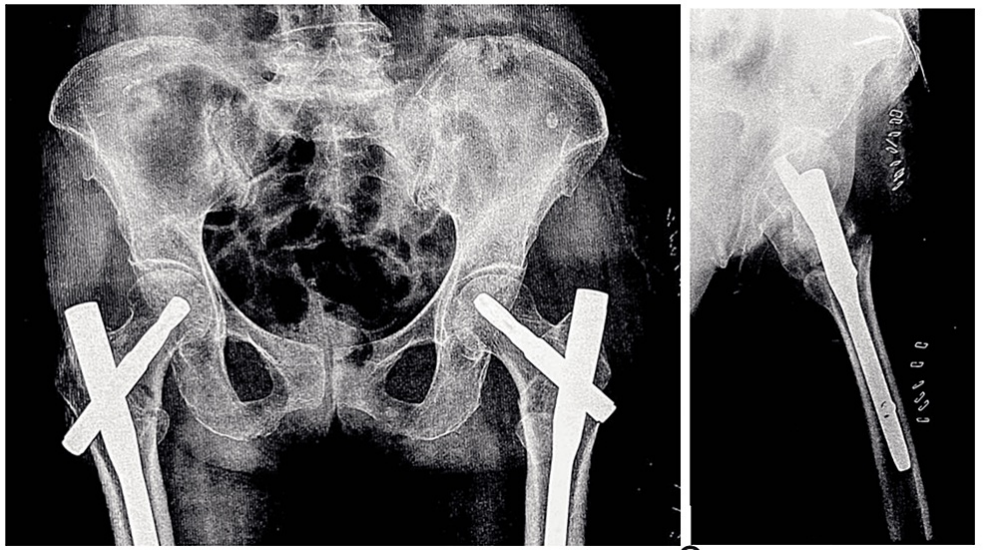
Plain radiograph of the pelvis with both hips showing operated cases of bilateral intertrochanteric fracture with in situ PFNA2 PFNA2: proximal femoral nail anti-rotation Asia

The patient underwent the closed reduction internal fixation surgical procedure using PFNA2 (Figure 2). After the surgery, the patient was released on postoperative day 14 and instructed to bear partial weight on the affected limb. The patient returned for a routine follow-up appointment after two weeks, and there were no notable incidents or complications.

**Figure 3:**
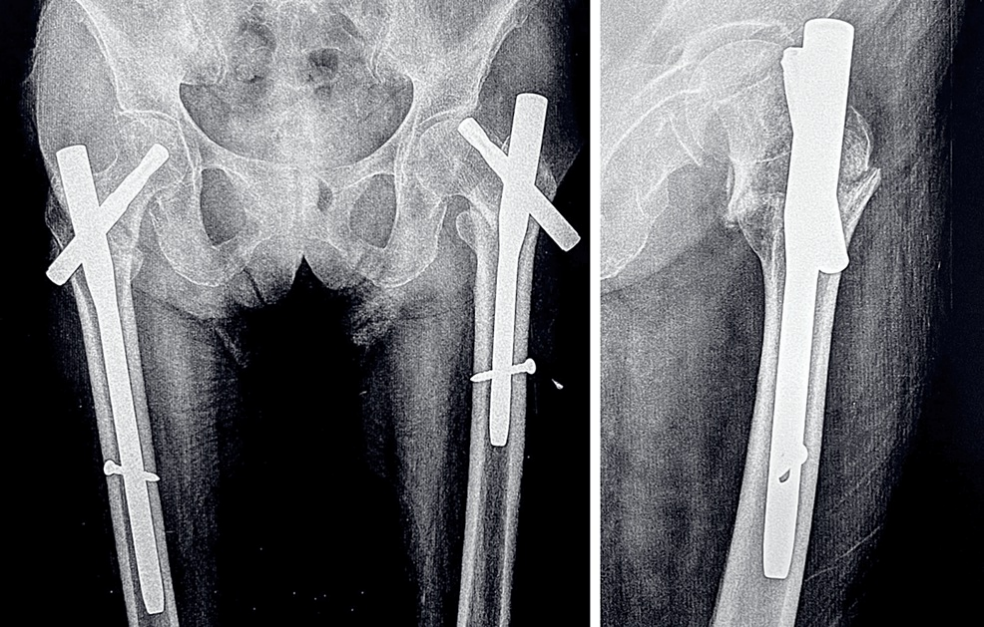
Plain radiograph of pelvis with both hips showing screw cut-out on the left side (postoperative day 15).

On postoperative day 21, the patient arrived at the outpatient department with discomfort in the left hip joint. Notably, there was no reported history of falling or experiencing any kind of physical injury. A follow-up radiograph revealed a posterior displacement of the blade of the left-sided short PFNA2 (Figure 3).

**Figure 4:**
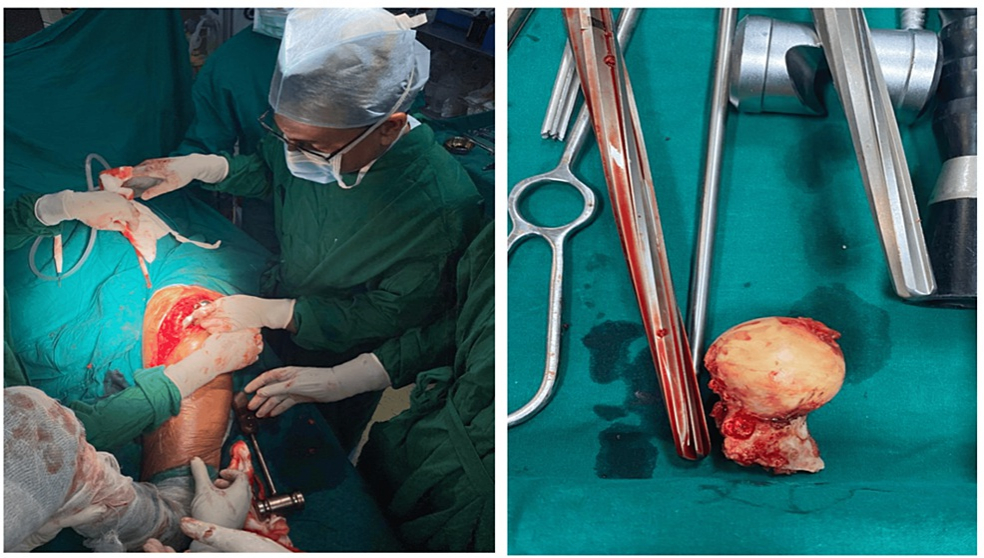
Intraoperative photographs of (a) the patient undergoing left bipolar hemiarthroplasty and (b) the femoral head

**Figure 5:**
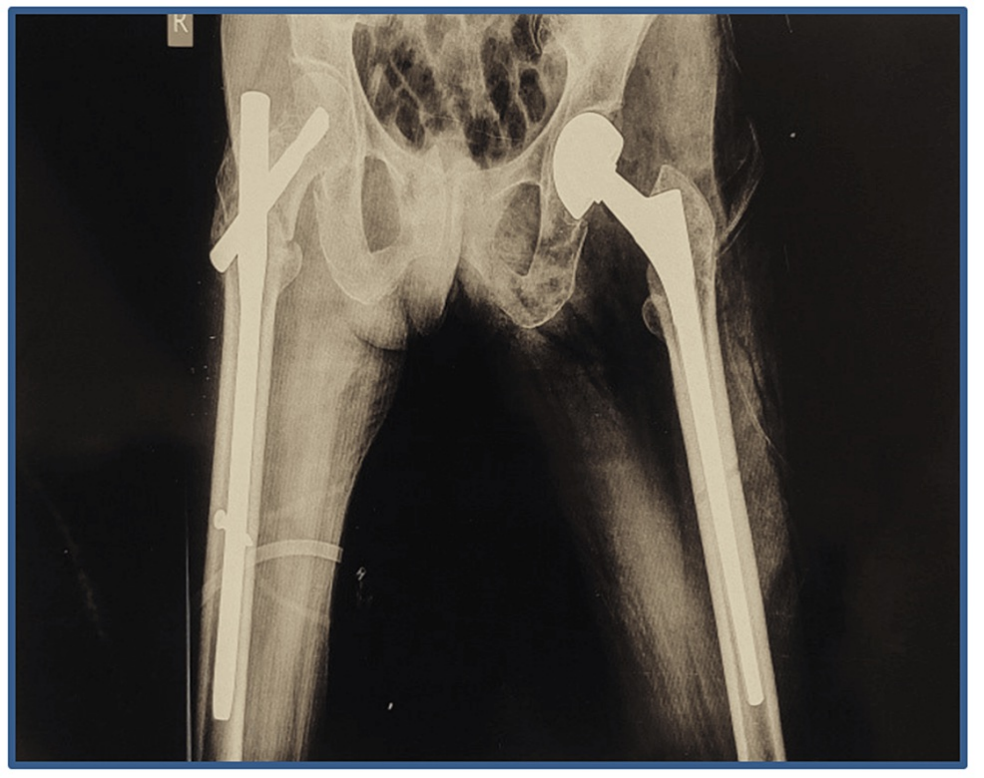
Postoperative X-ray of the pelvis with both hips anterior-posterior operated with bipolar hemiarthroplasty

A CT scan of the hip joints was done, which revealed no signs of union. Based on radiographic and clinical investigations, there were three potential causes of the implant failure on the patient’s left side. First, the path of the blade or nail may have been incorrect. Second, the surgeon may have failed to insert the lag screw within 5 mm of the anterior-posterior axis without the risk of penetration. Third, the temporary anchorage device (TAD) was under 20 mm, which can result in cut-outs, inability to bear weight after surgery, prevention of fracture impaction, trauma, or strenuous activity. The available options to manage the patient’s current condition were limited to uncemented or cemented bipolar hemiarthroplasty, total hip arthroplasty (THR), and revision PFN. Following a consultation with the patient, a surgical procedure known as left-sided bipolar hemiarthroplasty was performed (Figures 4, 5).

The patient’s pain was evaluated using a postoperative visual analog scale (VAS). The patient reported VAS scores of 5, 4, and 7 on postoperative days 2, 7, and 14, respectively. The patient underwent non-weight-bearing walking with the assistance of a walker for the first month after the operation. Subsequently, the patient transitioned to toe-touch walking and gradually progressed to full -weight-bearing walking without a walker. Nevertheless, the patient was prohibited from squatting.

## Discussion

Osteoporosis affects the entire skeletal system and is characterized by a decrease in BMD and a loss of bone structural integrity, resulting in an increased risk of developing fractures. As such, osteoporosis has emerged as a significant public health issue. Osteoporosis-related fractures are predicted to occur in 13%-30% of men and 30%-50% of women globally at some point in their lifetime [12]. The incidence of hip fractures in older adults, which are known to have serious implications for health and mortality rates, is possibly the most disastrous impact of osteoporosis. Adding to the intricacy of treatment is the fact that weakened bones damaged by osteoporosis and a frail blood supply increase the probability of fixation failure. In general, the incidence of fixation failure in hip fractures caused by osteoporosis ranges from 5% in pertrochanteric fractures to 15% and 41% in undisplaced and displaced fractures of the femoral neck, respectively.

The PFNA2 is an intramedullary fixation device that falls into the category of central fixation and provides considerable benefits in treating complicated osteoporotic fractures as compared to the nail-board technique. The prevalence of issues and failures following surgery varies depending on the adoption of different design principles. Femoral shaft fractures are distinct problems that may occur following intramedullary treatment of IT fractures due to the specific design characteristics of the chosen internal fixation method. Weight-bearing stress in patients may result in concentrated pressure on the distal intramedullary nail, locking nail, and nail junction, thereby causing long-term stress on these fracture areas. Elderly persons with osteoporosis often experience significant thinning of the cortical bone in the spine. The disadvantages of PFN include a high failure rate in increasing osteoporotic bone.

The most common complication of surgery for IT fractures is failure of the fixation in the femoral head and neck, which requires the reinsertion of a nail through the femoral head. Imprecise placement and inadequate depth of the spiral blade, as well as the quality of the bone, are critical factors that could lead to the severing of the head and neck of the screw. As stated by Al-Yassari et al., the placement of the spiral blade is the main factor that influences the stability of fixation; they suggested that the most advantageous placement for the spiral blade is at the center of the femoral head, positioned below its lower part [13]. The incidence of this complication is increased 5.9-fold when the screw is placed at a position two-thirds of the distance from the top of the head and neck. The depth of the spiral blade directly affects the precision and screw penetration in the cranial and cervical regions.

Although the failure rate of internal fixation is not directly correlated with the severity of osteoporosis, several studies have reported that no fixation device can successfully produce a stable internal fixation and promote early activity in patients with severe osteoporosis [14]. According to research by Bonnaire et al., the lack of success in surgical techniques such as dynamic hip screws, gamma nails, and PFN postoperative cutting is overwhelmingly linked to osteoporosis; in their study, they report a substantial correlation between a lower bone mineral density (i.e., <0.6 g/cm^3^) and increased risk of fixation failure [15]. Therefore, if preoperative and postoperative protocols do not incorporate the comprehensive treatment of osteoporosis, participating in functional activities after surgery would not be appropriate. Moreover, neglecting to address the management of rotation in the femoral neck and safeguarding against fractures using external fixation could lead to surgical complications and problems with the internal fixation apparatus. Such complications include the screws piercing the femoral head, severing of the neck, harm to the hip joint, and loosening of the nail-all of which can result in fixation failure and non-union.

The available options following a failed PFN procedure include refixation, bipolar hemiarthroplasty, and THR. In the present case, bipolar hemiarthroplasty was chosen owing to its lower rate of dislocation with bipolar as compared to THR in cases of traumatic proximal femoral fracture [16]. THR is often complicated by a high dislocation rate. Several studies have favored the use of bipolar implants in healthy older patients [17] due to their low dislocation rate and favorable functional results. However, it is important to note that this surgery is also associated with a high revision rate, which might complicate the overall outcome. Several distinct issues might arise when converting unsuccessful internal treatment of IT fractures to hip arthroplasty. The incidence of complications in conversion total hip arthroplasties (THAs) exceeds that observed in primary THAs. The risk of infection is often increased in places that have previously undergone surgery and when additional hardware is introduced [17].

## Conclusions

The main factors contributing to internal fixation failure include improper implant placement, inadequate tip apex distance, pronounced osteoporosis, and incorrect positioning of the helical blade. The degree of osteoporosis is closely correlated with the occurrence of internal fixation failure. If internal fixation is unsuccessful, it is recommended to consider either replacing the joint with an artificial one or re-fixing it while considering the specific cause of the failure and the unique circumstances of the patient, as this approach will maximize the chances of favorable treatment results. We recommend tailoring the therapy based on the patient’s age, level of physical activity, and bone density.
